# Efficiency of Different Disinfectants on *Bacillus cereus* Sensu Stricto Biofilms on Stainless-Steel Surfaces in Contact With Milk

**DOI:** 10.3389/fmicb.2018.02934

**Published:** 2018-11-28

**Authors:** Higor Oliveira Silva, Joyce Aparecida Santos Lima, Carlos Eduardo Gamero Aguilar, Gabriel Augusto Marques Rossi, Luis Antonio Mathias, Ana Maria Centola Vidal

**Affiliations:** ^1^Department of Preventive Veterinary Medicine and Animal Reproduction, School of Agrarian and Veterinarian Sciences, São Paulo State University, São Paulo, Brazil; ^2^Department of Veterinary Medicine, School of Animal Science and Food Engineering, University of São Paulo, São Paulo, Brazil

**Keywords:** peracetic acid, sodium hypochlorite, recontamination, food safety, biofilm formation

## Abstract

The species of the *Bacillus cereus* group have the ability to adhere to and form biofilms on solid surfaces, including stainless steel, a material widely used in food industries. Biofilms allow for recontamination during food processing, and the “clean-in-place” (CIP) system is largely used by industries to control them. This study thus proposes to evaluate the efficacy of peracetic acid and sodium hypochlorite against biofilms induced on stainless-steel surfaces. The SAMN07414939 isolate (BioProject PRJNA390851), a recognized biofilm producer, was selected for biofilm induction on AISI 304 stainless steel. Biofilm induction was performed and classified into three categories: TCP (Tindalized, Contaminated, and Pasteurized milk), TCS (Tindalized milk Contaminated with Spores), and TCV (Tindalized milk Contaminated with Vegetative cells). Subsequently, the coupons were sanitized simulating a CIP procedure, on a pilot scale, using alkaline and acid solutions followed by disinfectants (peracetic acid and sodium hypochlorite). Microorganism adhesion on the surfaces reached 6.3 × 10^5^ to 3.1 × 10^7^ CFU/cm^-2^. Results did not show significant differences (*p* > 0.05) for surface adhesion between the three tested categories (TCP, TCS, and TCV) or (*p* > 0.05) between the two disinfectants (peracetic acid and sodium hypochlorite). Microbial populations adhered to the stainless-steel coupons are equally reduced after treatment with peracetic acid and sodium hypochlorite, with no differences in the control of *B. cereus* *s.s.* biofilms on AISI 304 stainless-steel surfaces.

## Introduction

Biofilm formation is a complex process consisting of a string of molecular and physiological events that take place throughout several stages including adherence, formation of microcolonies, tridimensional structuring, and maturation (Watnick and Kolter, 2000). Bacteria of the *Bacillus cereus* group have the ability to adhere to and form biofilms on solid surfaces such as stainless steel (Salustiano et al., 2009; Kumari and Sarkar, 2016). In the industry, milk residues may occasionally persist on the surface of stainless-steel equipment forming a thin layer, rich in nutrients, called conditioning film that renders these surfaces more prone to bacterial adhesion and the consequent formation of biofilms (Machado, 2005).

Reports of the presence of *B. cereus* microorganisms in commercial products are frequent (Vidal-Martins et al., 2005; Montanhini et al., 2015; Vidal et al., 2016), and biofilms play an essential role in the persistence of these microorganisms in processing lines. Biofilms have an extracellular matrix that constitutes a stable structure that protects bacteria against the action of sanitizing agents, making their control more difficult and allowing for persistent recontamination of food products (Simões et al., 2006; Salustiano et al., 2009; Majed et al., 2016). Another mechanism related to the biofilm persistence is considered the different gene expression in the multicellular population, cause some cells enter in a dormant or persistent state, manifesting a non-inherited resistance or tolerance to different antimicrobials (Wood et al., 2013; Singh et al., 2017).

The “clean-in-place” (CIP) system is commonly employed by industries to control biofilms in milk processing lines. It is a sanitization procedure that includes the regular cleaning of pipes and equipment by using acid and alkaline solutions applied at high temperatures (Bremer et al., 2006). It is used essentially to ensure the disinfection of clean surfaces and the elimination of organic residue through the action of sanitizers in hard-to-reach places (Shi and Zhu, 2009). However, product contamination and deterioration caused by biofilms are still recurring problems (Ostrov et al., 2016).

Microorganisms of the *B. cereus* group are among the most important deteriorating agents of the milk production chain and are also among those involved in foodborne diseases. Because they compromise the quality and microbiological safety of milk and dairy products, they represent a major concern for the dairy industry (Simões et al., 2010; Kumari and Sarkar, 2016).

In the last decade, the scientific community has been increasingly interested in research on biofilm formation by microorganisms of the *B. cereus* group (Majed et al., 2016). However, few studies have investigated the efficiency of processes to combat biofilms formed by *B. cereus* bacteria, mainly those adhered to stainless-steel surfaces. By contrast, these studies clearly show the importance of determining the efficiency of solutions and sanitizers used in CIP procedures for the removal of bacteria linked to stainless-steel surfaces (Parkar et al., 2003; Bremer et al., 2006).

The present study aimed to evaluate the efficiency of two types of disinfectants (peracetic acid and sodium hypochlorite) used in the CIP system on biofilms induced by spores and vegetative cells of *Bacillus cereus s.s.* formed on stainless-steel surfaces that were in contact with experimentally tindalized milk.

## Materials and Methods

**FIGURE 1:**
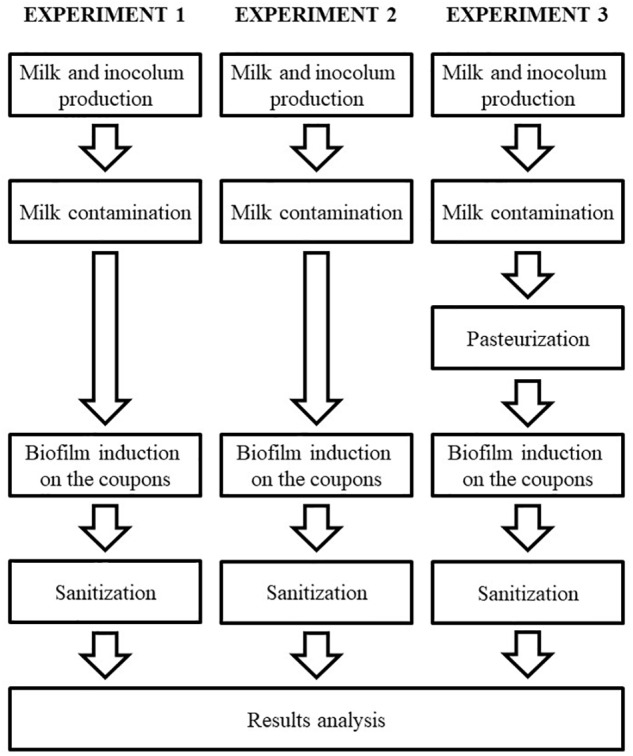
Flowchart of the experimental design, describing all the steps of this study.

A strain of *Bacillus cereus s.s.* previously genetically sequenced (SAMN07414939 – BioProject PRJNA 390851) and phenotypically considered as biofilm producer was selected for the present study, which was performed in three separated experiments (Figure 1), each with three repetitions, as described in details in the following sessions.

Initially, the milk used in the study was tindalized, to allow the total elimination possible of sporulated bacteria. Then, the milk was experimentally contaminated with the strain of *B. cereus s.s.* selected. Coupons of stainless steel were submerged into the contaminated milk for 10 h to allow the biofilm formation on its surface. Posteriorly, the coupons were sanitized and the efficiency of the sanitization was evaluated based on the counts of the bacterial population adhered before and after the process.

### Experiment 1

An inoculum of vegetative cells of *B. cereus s.s.* (SAMN07414939 – BioProject PRJNA 390851) was produced to contaminate the tindalized milk. For this, 10 μL of the isolate kept in stock was transferred to test tubes containing 5mL of Brain-Heart Infusion Broth (BHI) and incubated at 30°C for 12 h, performing the count of the population in 1 mL of the culture at the time of use.

To assure that the biofilm production was restricted to the vegetative cells of the *B. cereus s.s.* incubated a milk free of contamination was prepared. For this, 30 liters of raw milk were submitted to the tindalization process.

The tindalization was performed in the dairy industry of the City Campus Fernando Costa (PUSP-FC), located in Pirassununga, SP, Brazil. The equipment used include the yogurt-making machine (composed by a Fermentation tank made by stainless steel – Mec Milk^®^), with temperature control, coupled by tubes to a pasteurizer. Previously, the equipments were mechanically cleaned and sanitized with sodium hydroxide, nitric acid, and peracetic acid with concentrations at 0.05% at 25°C for 1 h. Swabs were collected before and after the sanitization in order to evaluate the efficiency of the process in the equipment and pipes.

The tindalization was performed with a series of heatings: first, the milk was heated at 80°C for 10 min, then cooled at 30°C for 30 min, and then re-heated at 95°C for 20 min. The entire process was repeated three times (Kim et al., 2012, adapted).

Thirty liters of raw milk were tindalized and pumped through the pipes from the raw-milk-receiving tank to the pasteurization machine. Of these, 10 liters were pasteurized and packed in polyethylene bags with a capacity of 1 liter. Posteriorly, the Tindalized Milk (TM) was transported to the Laboratory of Quality of Animal Products (Quali-POA) and stored under refrigeration until the moment of analysis.

**FIGURE 2:**
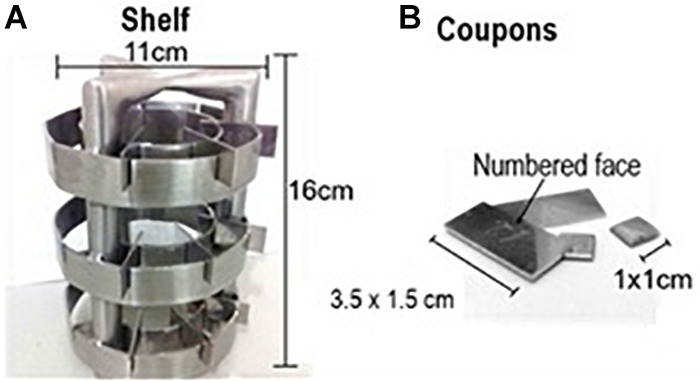
Experimental prototype used in this study, made of AISI 304 stainless steel with sanitary finishing.

An experimental prototype (Figure 2) was produced in stainless steel AISI 304, with coupons whose dimensions were 3.5 × 1.5 cm and 1 × 1 cm (coupons A and B, respectively), and prepared for the adhesion of *B. cereus s.s.*, and a shelf with the capacity of 30 coupons (Oliveira et al., 2010, adapted). The coupons A were produced to perform the bacterial counts, presenting an area more adequate to the quantification so that the adhered population would not be underestimated. The coupons B were produced exclusively to perform the topography analysis after the biofilm induction, adapted to the size and capacity of the Scanning Electron Microscopy cannon. The prototype was previously sanitized by mechanical abrasion with sponge and neutral detergent, followed by a 1% sodium hydroxide, immersion in 70% alcohol and autoclaving (Ribeiro-Furtini, 2005).

To create a conditioning film in the surfaces of the coupons of stainless steel, similar to that found on food industries, 21 coupons (18 coupons A and 3 coupons B) were arranged in the shelf and were totally submerged in 1 liter of TM inside of a sterile beaker under stirring for 1 h at room temperature. The entire process was conducted in a biosafety cabinet. Posteriorly, to ensure the sterility after the procedure three coupons A were subjected to bacterial quantification with surface swabs. The coupons were transferred aseptically to sterile petri dishes with filter paper with the sampling surface turned up and dried at 60°C for 2 h.

In sequence, the milk contamination was performed transferring 1 liter of TM to a sterile beaker containing a prototype with 18 coupons (15 coupons A and 3 coupons B) to induce the bacterial adhesion. 2 mL of the inoculum were added to this milk at room temperature, remaining incubated under agitation for 10 h to induce the adhesion of the *B. cereus s.s.*

### Experiment 2

Ten micro liter of the culture of *B. cereus s.s.* in stock were transferred to the Tryptone Soy Agar (TSA) and incubated for 7 days at 30°C to induce the spore production. In sequence, the agar surface was washed with 5 mL of sterile distilled water. The mixture was transferred to sterile tubes and then centrifuged for 20 min. The supernatant was discarded and resuspended with sterile distilled water, followed by a new centrifugation, repeating the process three times.

Posteriorly, the suspension was submitted to a thermal shock at 80°C for 1 min followed by a cooling for 1 min to inactivate the vegetative cells (Giffel et al., 1997). The absence of vegetative cells was confirmed by the Wirtz-Conklin staining (Bier, 1975) and the count of the *B. cereus s.s.* was performed using 1 mL of the culture at the time of use.

In this experiment the tindalized milk free of contamination was also used, as described on the Experiment 1. The TM was transported to the laboratory Quali-POA and refrigerated until the moment of the analysis. Then, the contamination was performed by adding 2 mL of the inoculum in one liter of the TM and the rest of the experiment followed the same steps of the Experiment 1.

### Experiment 3

For the third experiment was used the same inoculum produced in the Experiment 1. After the tindalization of the raw milk, 10 liters of TM were pumped through to the raw-milk-receiving tank, where 15 mL of the inoculum of vegetative cells of *B. cereus s.s.* were added. The TM (at 30°C) was homogenized, pasteurized and packed in polyethylene bags. After this, the milk was transported to the laboratory, where was refrigerated until the moment of the next analysis and the rest of the experiment followed the same steps of the Experiment 1.

### Sanitization of the Stainless Steel Coupons by the Clean-In-Place (CIP) System, Simulated in a Pilot Scale

The same sanitization process was repeated to three experiments.

**FIGURE 3:**
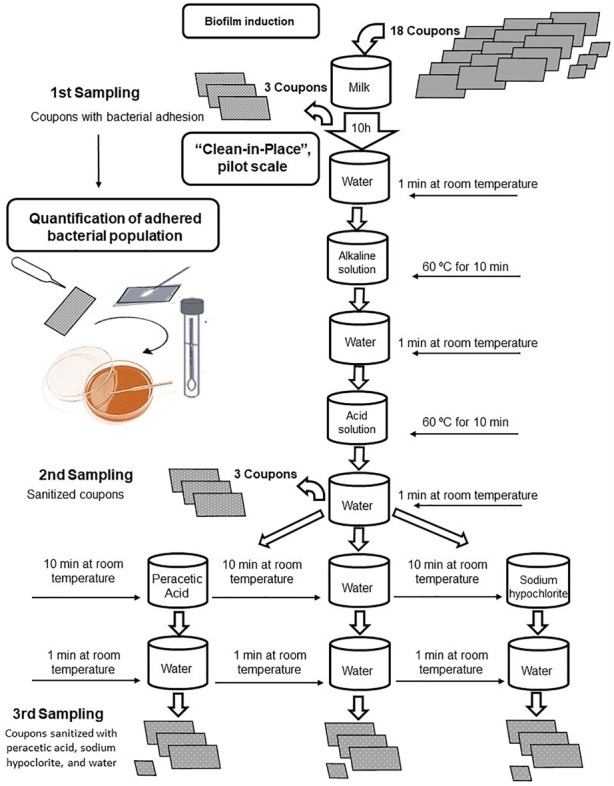
Flowchart of the clean-in-place procedure applied to stainless-steel coupons with *Bacillus cereus s.s.* biofilm adhesion.

After the incubation for 10 h, the prototype containing 15 coupons (12 coupons A and 3 coupons B) with biofilm adhered was submitted to the sanitization process. This stage was composed by seven steps, each involving one solution normally used in the dairy industry, as showed in Figure 3. The experimental prototype was submerged in the beakers, sterilized, and kept under agitation and heating on a magnetic stirrer.

The sanitization started with water at room temperature for 1 min, followed by an alkaline solution (2% sodium hydroxide) at 60°C for 10 min, sterile distilled water at room temperature for 1 min, acid solution (2% nitric acid) at 60°C for 10 min, and washed again with sterile distilled water at room temperature. In this step, the second lot of samples of the coupons A were collected.

The CIP process was completed after the sanitization with a 0.1% solution of peracetic acid and 0.02% sodium hypochlorite, both at room temperature for 10 min, followed by the rinse with sterile distilled water at room temperature for 1 min. For the negative control, the sanitization process was performed using only water, without the aid of the sanitizers.

At the end of this step, the third and last proceeding of collect of the coupons A was performed, in triplicates, and the coupons B, individually reserved in sterile Petri dishes with filter paper, where remained at 30°C for 24 h.

### Quantification of the Bacterial Population and the Adhesion to the Coupon Surfaces

To quantify the bacterial population aliquots of 1 mL of the milk and surface swabs were collected. Serial dilutions were performed using 0.1% sterile peptone water and then 0.1 mL of the mixtures were transferred to TSA and Mannitol Egg Yolk Polymyxin Agar (MYP) using the surface coating technique. The plates were incubated at 30°C for 24–48 h.

To quantify the population adhered to the coupons, they were washed and then swabs were smeared on the surfaces.

The first lot of samples was washed with phosphate buffer to remove the residues of milk, followed by additional washes with sterile distilled water, saline solution of tween 80 peptone (H_2_Osp-Tween) and sterile distilled water again. On the second and third lot of samples the coupons were washed with sterile distilled water H_2_Osp-Tween and sterile distilled water again.

Three coupons were collected from each lot, using a sterile swab for each coupon, which posteriorly were submerged into tubes containing 9 mL of 0.1% sterile peptone water. Serial dilutions were performed, following by plating in TSA and MYP at 30°C for 24–48 h.

### Topographic Analysis of the Coupons of Stainless Steel Surfaces

After incubation for 24 h, the coupons B were taken to the Electronic Microscopy Laboratory at the School of Agrarian and Veterinarian Sciences of the Sao Paulo State University (UNESP), located at Jaboticabal, SP, Brazil. The coupons were immersed on a fixing solution (Karnovsky modified: 2.5% glutaraldehyde, 2.5% formaldehyde in 0.05 M sodium cacodylate buffer at pH 7.2 and 0.001 M of CaC_12_) during at least 72 h.

Posteriorly the coupons were washed with phosphate buffer, fixed overnight in 1% osmium tetroxide, dehydrated using an ethanol gradient (25, 50, 70, 90, and 100%), dried at critical point and golden metallized (Bossola and Russell, 1998). Then, the topographic images of the coupons surfaces were obtained using a Zeiss EVO MA-10 electronic microscopy.

### Statistical Analyses

Population count results were analyzed via ANOVA and T test at the 5% significance level.

## Results

In the experimental contamination, 2.4 × 10^9^ to 7.2 × 10^10^ CFU.L^-1^ milk were inoculated, resulting in populations of 1.1 × 10^8^ and 7.6 × 10^9^ CFU.mL^-1^ milk at the end of 10 h.

Biofilm formation and adhesion were observed in the three categories evaluated – TCP, TCS, and TCV –, as shown in Table 1. The average *B. cereus s.s.* populations in biofilms adhered to A coupons ranged from 6.3 × 10^5^ to 3.1 × 10^7^ CFU/cm^2^, with no significant differences (*p* > 0.05) between the three categories under study; i.e., there was no difference between adhesion of spores and vegetative cells.

**Table 1:** Average counts of *Bacillus cereus s.s.* in biofilms adhered to AISI 304 stainless-steel surfaces in contact with tindalized, contaminated, and pasteurized milk; tindalized milk contaminated with spores; and tindalized milk contaminated with vegetative cells.

Treatment	CFU/cm^2^				
	CA	CC	NC	CSP	CSH
TCP	3.4 × 10^7^ ± 3,07	1.1 × 10^7^ ± 8,39	8.1 × 10^6^ ± 4,36	5.8 × 10^6^ ± 4,86	4.6 × 10^6^ ± 4,47
TCS	6.3 × 10^5^ ± 2,72	3.6 × 10^5^ ± 5,25	1.5 × 10^5^ ± 7,20	2.5 × 10^5^ ± 2,40	3.0 × 10^4^ ± 2,69
TCV	3.1 × 10^7^ ± 1,93	8.1 × 10^6^ ± 2,54	8.7 × 10^6^ ± 4,99	8.5 × 10^5^ ± 8,05	5.5 × 10^4^ ± 5,30

*TCP, tindalized, contaminated, and pasteurized milk; TCS, tindalized milk contaminated with spores; TCV, tindalized milk contaminated with vegetative cells; CA, coupon with adhesion; CC, cleaned coupon; NC, negative control; CSP, treatment with peracetic acid; CSH, treatment with sodium hypochlorite. The counts of *Bacillus cereus s.s.* in biofilms are presented as mean ± standard deviation*.

**FIGURE 4:**
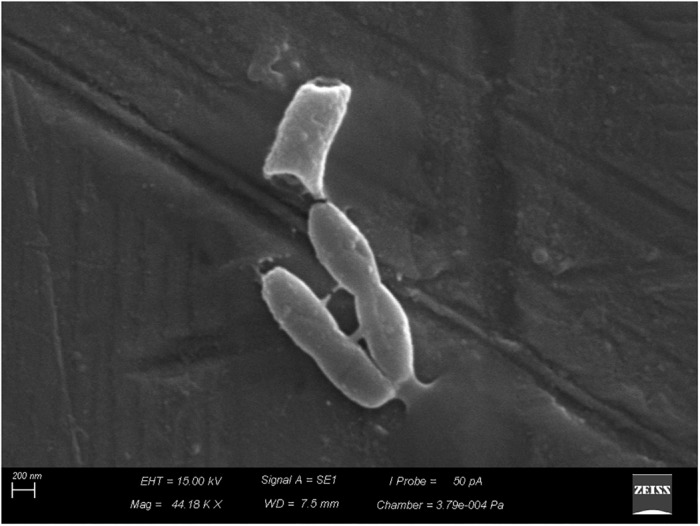
Adhesion of vegetative cells of *Bacillus cereus s.s.* on AISI 304 stainless-steel surface.

**FIGURE 5:**
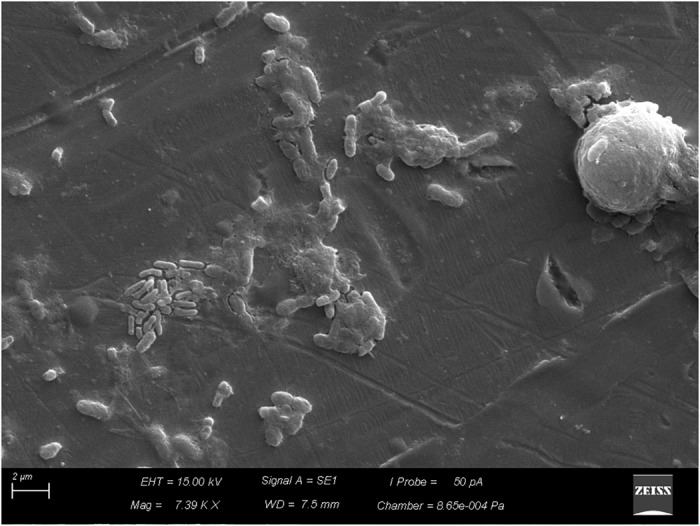
Production of supposed extracellular matrix by vegetative cells of *Bacillus cereus s.s.* on AISI 304 stainless-steel surface.

Scanning electron microscopy revealed adhesion of vegetative cells of *B. cereus s.s.* to the stainless-steel surface (Figure 4) and production of supposed extracellular matrix (Figure 5).

**FIGURE 6:**
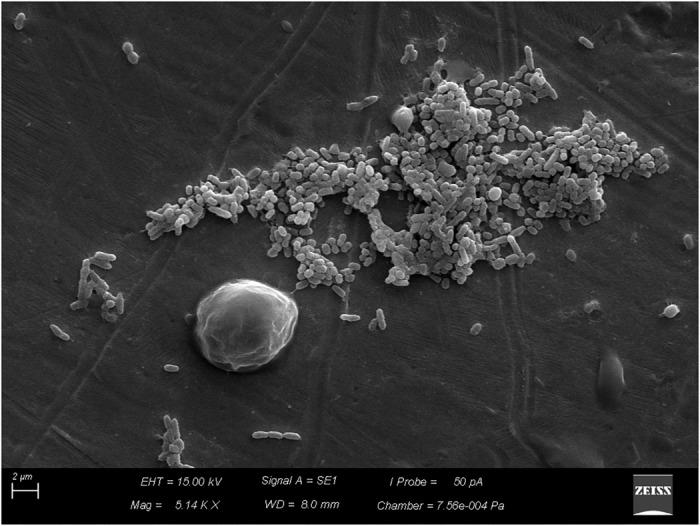
Beginning of the process of biofilm formation by vegetative cells of *Bacillus cereus* *s.s.* on AISI 304 stainless-steel surface with cell proliferation and formation of three dimensional structure.

**FIGURE 7:**
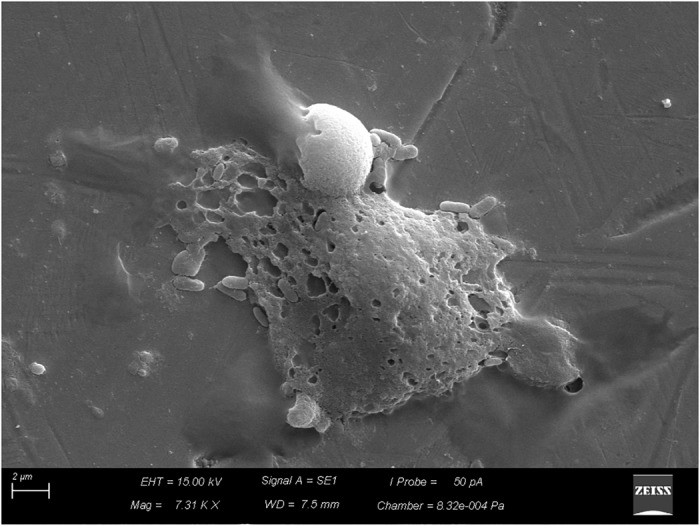
Biofilm already structured by vegetative cells of *Bacillus cereus* *s.s.* on AISI 304 stainless-steel surface.

**FIGURE 8:**
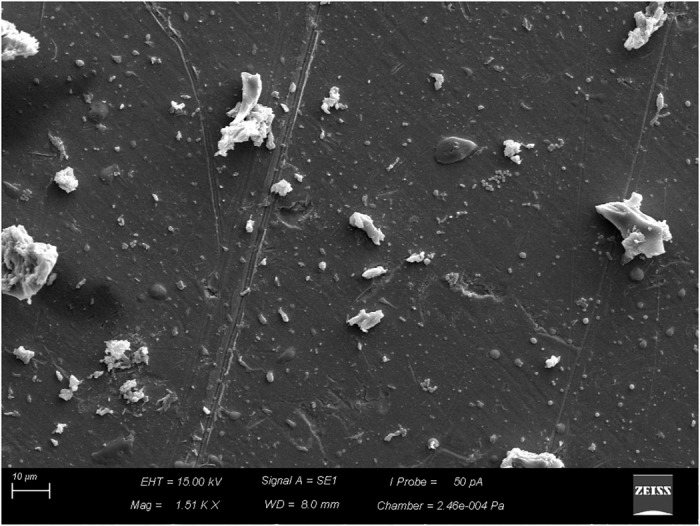
Residues of milk and other components (organic and inorganic) on AISI 304 stainless-steel surface, obtained from tindalized milk.

After the coupons were sanitized by the CIP system, simulated on a pilot scale, despite the reduction of *B. cereus* counts, which varied between 3.6 × 10^5^ and 1.1 × 10^7^ CFU/cm^2^, biofilm removal was not complete with the disinfectants tested. The biofilms evolved to more-structured stages (Figures 6, 7), and organic residues from milk were observed (Figure 8).

The *B. cereus* counts in coupons cleaned with water only, peracetic acid, and sodium hypochlorite ranged from 1.5 × 10^5^ to 8.7 × 10^6^, 2.5 × 10^5^ to 8.5 × 10^6^, and 3.0 × 10^4^ to 4.6 × 10^6^, respectively. No statistically significant difference was observed (*p* > 0.05) between the populations after the sanitizers were applied and after the simple use of water in the final stage of the CIP procedure, which was simulated on a pilot scale.

## Discussion

Research points to a greater adhesion capacity of spores as compared with vegetative cells (Faille et al., 2001; Branda et al., 2005; Jang et al., 2006). However, this was not observed in the results of the current study, where no significant difference was detected (*p* > 0.05) between adhesion and colonization in the studied categories.

This greater adhesion may not be related to multispecies biofilms, since spores of *B. cereus* microorganisms have a competitive advantage over other species due to the reduction of the competing load resulting from the thermal treatment applied to the milk. In the present case, where biofilm was induced in environments (due to their synergistic activities) totally restricted to *B. cereus s.s.* spores, adhesion at different intensities was not evident (Sharma and Anand, 2002).

Despite the lack of differences, the initial adhesion of spores, favored by the conditioning film, where the spores germinate and vegetative cells can synthesize the matrix components, multiply and form a biofilm, is worrying because of its resistance to chemical agents and thermal treatments. Spores are resistant to disinfection and other inactivation methods, compared with vegetative cells (Young and Setlow, 2003). Previous studies have shown that peracetic acid does not reduce the spores of *Bacillus* sp. to undetectable levels, and it is considered even less effective in the decontamination of spores when compared with chlorine dioxide and ozone (Jang et al., 2006).

This protection may be associated with several factors, including enzymatic complementation and the organized spatial distribution of cells in the biofilm (Burmølle et al., 2006). The second factor was observed in this study (Figures 6, 7).

The sanitization process is divided into cleaning and disinfection, whose purpose is to remove organic and mineral residues adhered to the surfaces, besides eliminating pathogenic microorganisms and reducing the microbial load to levels considered safe, respectively (Rossi, 2008). However, we observed that in spite of the reduction of microbial load, the biofilms evolved to more-structured stages (Figures 6, 7), and milk residues were detected (Figure 8), demonstrating that, in addition to disinfectants, the acid and alkaline components also play an important limiting the factors for conditioning films.

In this study, even after sanitization, there was microorganism adhesion on the coupons’ surfaces. Other studies have demonstrated that bacteria can survive and produce biofilms inside pipes even after a properly applied CIP procedure (Shi and Zhu, 2009). In China, Zhou et al. (2008) described that the powder milk contained less *B. cereus* isolates than raw milk, which can be associated to the presence of spores on the raw material and biofilms formed inside the pipes that are resistant to heat, drying and CIP procedure.

In this sense, the results of this study agreed with the study of Ribeiro et al. (2017) demonstrating that the conditioning of their functions is a determining factor in the adhesion degree of *Bacillus cereus* biofilms.

The milk residues render surfaces more prone to bacterial adhesion and consequent biofilm formation (Machado, 2005), as Surface conditioning changes its physical-chemical properties, may affect the order of adhesion events and biofilm formation and increase microbial fixation (Simões et al., 2010).

Biofilms associated with dairy processing industries are favored by the presence of conditioning films, generated mostly by residual milk, which allows for the accumulation of organic and inorganic milk compounds (Ribeiro, 2015). In addition, disinfectants are less effective when there are residues of organic materials such as fats, sugars and proteins (Wirtanen and Salo, 2004). Therefore, it is essential that hygiene procedures are conducted using detergents to remove residues of organic and inorganic food associated with sanitization by the use of physical or chemical agents to control microorganisms (Salustiano et al., 2010).

In this way, to prevent and combat the contamination of equipment by biofilms, especially given the trends of use of increasingly complex and automated equipment and plants, increasingly rigorous microbiological parameters must be adopted and required (Pasvolsky et al., 2014). Even under the condition of using automatic CIP plants, a constant microbiological control over the efficacy of the sanitization should be implemented in the dairy industries to ensure safe products (Kukhtyn et al., 2017).

Peracetic acid is considered effective against spores (Mohan et al., 2009; Buhr et al., 2013). The research has shown that despite their resistance, peracetic acid can inactivate spores. Apparently, this mechanism does not occur through damage to the DNA, but probably as a result of alterations in the external layers of spores, more specifically the interim layer, such that when they germinate the membrane will break (Buhr et al., 2013; Park et al., 2014; Leggett et al., 2016). However, the mechanism of spore inactivation by peracetic acid is not fully known.

To improve the ability to destroy and remove biofilm from food processing facilities, the use of combined sanitizing treatments with other methods may be more effective than the use of any factor alone (Ban and Kang, 2016). It is possible that higher concentrations of peracetic acid are more effective against spores; however, using high concentrations of sanitizers in industrial environments is a challenge, as it may favor the corrosion process (Jang et al., 2006).

To improve the ability to destroy and remove biofilm from food processing facilities, the use of combined sanitizing treatments with other methods may be more effective than the use of any factor alone (Ban and Kang, 2016). Previous studies have shown that the bactericidal effect of peracetic acid is much more effective when combined with gaseous components such as carbon monoxide (White et al., 2006). The decontamination of spores with chlorine dioxide and ozone is more effective against spores of *Bacillus* sp., Because peracetic acid does not reduce to undetectable levels (Jang et al., 2006).

The combined treatment of disinfectant and steam is a very promising alternative technology to control biofilm cells from non-spore-forming pathogens. However, spore efficiency is still studied (Ban and Kang, 2016). In addition to these studies, a number of other approaches can be used to control biofilms in the dairy industry such as altering the chemical nature of the surface to prevent cell binding, treating surfaces with antimicrobial agents, and optimizing equipment design, processes and CIP cleaning regimes remain particularly important (Gopal et al., 2015).

The membrane of spores also provides resistance to hypochlorite (Sabli et al., 1996). In contrast, spores treated with hypochlorite have difficulty germinating, although those resisting treatments with hypochlorite do not present visible damage to the DNA (Young and Setlow, 2003). Both the nutrient receptors and the receptors of lytic enzymes from the cellular cortex of spores are possibly greatly damaged by treatment with hypochlorite. The authors believe hypochlorite damages the membranes by oxidizing fatty acids or by oxidizing membrane proteins, which can both occur (Young and Setlow, 2003).

Results point to a lack of differences between the use of the sanitizers applied in this study and the use of water during the final stage of the CIP procedure. This may be a consequence of the vortex effect caused by agitation, which is probably effective in the removal of microorganisms with weak adhesion.

The stage of bacterial adhesion is considered fundamental; without it, the process does not evolve (Wirtanen et al., 1996; Klemm et al., 2010). It is also the most vulnerable to the action of disinfectants (Ghigo, 2003; Webb et al., 2003).

A characteristic of CIP operations is their variable effectiveness in eliminating planktonic bacteria or surface-adhered biofilms (Faille et al., 2001; Marchand et al., 2012). Studies show that even using modern washing agents and disinfectants in the sanitization of dairy equipment, the equipment is not sterile after standard sanitization (Kukhtyn et al., 2017). This variability is due to an array of interfering factors; e.g., nature, time, and composition of biofilm; composition, concentration, time, and temperature of the cleaning agent; turbulence of the cleaning solution; among others. For this reason, the ideal CIP regime may vary across processing industries and also in specific points over time, in a given plant (Marchand et al., 2012).

Biofilm formation by *B. cereus s.s.* was not influenced by pasteurization in the study conditions, demonstrating that once installed in the processing line, biofilms can mature and disperse, colonizing also pasteurizing and packing machines (Eneroth et al., 2001; Faille et al., 2014).

*Bacillus cereus s.s.* survives pasteurization because of sporulation, and after germination, the cells are free from competition with other vegetative cells (Sharma and Anand, 2002). Although the biofilms formed by more than one bacterial species show more metabolic advantages in natural environments, biofilms formed by only one species of bacterium typically colonize the substrates and surfaces of equipment in dairy industries more effectively (Parkar et al., 2003).

## Conclusion

Peracetic acid and sodium hypochlorite were not effective in removing the *B. cereus s.s.* biofilms formed on stainless-steel coupons submerged in milk, with no difference from the same sanitization process performed without the use of disinfectants. These results are of extreme importance for dairy industries to adopt the use of efficient disinfectants in biofilm removal.

## Author Contributions

HS, GR, and CA performed the experimental stages. HS and JL wrote the article. JL wrote, adapted to the guidelines, reviewed, and finalized the article. LM performed the statistical analyzes. AV supervised all the steps, reviewed, and corrected the article.

## Conflict of Interest Statement

The authors declare that the research was conducted in the absence of any commercial or financial relationships that could be construed as a potential conflict of interest.
